# Rupture of the Gall-Bladder

**Published:** 1857-12

**Authors:** 


*Rupture of the Gall-Bladder.* Reported to the Medical Society of the County of Albany, by S. H. FREEMAN, M. D., and communicated to the Boston Medical and Surgical Journal, by LEVI MOORE, M. D., Secretary.

The patient was a gentleman retired from business, of vigorous constitution, florid complexion, and 65 years of age. He had usually enjoyed good health and spirits, with the exception of occasional attacks of cardialgia and other minor symptoms of dyspepsia.

I was first called on the 15th of October, 1857, to visit Mr. K., who was supposed to be suffering from an ordinary attack of colic. Found him vomiting and suffering occasional paroxysms of spasmodic pain, which he referred to the pit of the stomach and to the region of the umbilicus. There was no tumefaction of the bowels, nor tenderness on pressure. The tongue was slightly coated, brown, moist; pulse 80, full and regular. The vomiting and pain were soon relieved by the immediate administration of a dose of dilute chloric ether, followed by the exhibition of small doses of calomel and opium. He enjoyed a comfortable night, and the following morning had a free bilious evacuation from the bowels.

On my second visit the next day, at noon, I found him sitting in his easy chair, quite comfortable; and, indeed, he expressed himself "perfectly well." During the afternoon, however, he had a return of violent intestinal pain and vomiting, discharging with the contents of the stomach, about a pint of grumous blood, which was followed soon after by a copious bloody passage. He was, again, soon relieved, and had no return of farther unpleasant symptoms, with the exception of occasional paroxysms of pain, until a fortnight from the time of the first attack, when I was hastily summoned, at night, to visit him. I then found him *in articulo mortis*—the surface of his body was covered with cold perspiration, and though he conversed intelligently and calmly, his pulse, which the evening previous was perfectly normal, was now rapid, irregular and feeble; and in half an hour from the time I entered his room, he gently breathed his last.

*Autopsy*, fifty-six hours after death.

On opening the cavity of the abdomen, there appeared a mixture of sanguineous fluid and bile, amounting, by estimate, to three or four pints. On farther examination, *eleven* large biliary calculi (resembling chestnuts in size and shape) were found in the peritoneal cavity and in the gall-bladder. The gall-bladder was adherent to the duodenum and transverse colon. Its cavity was more than double the normal capacity, and its walls were much thickened. The *ductus choledochus communis* was greatly enlarged, and about midway between the fundus and the duct there was a free opening, about an inch and a half in diameter, through which the contents of the bladder had escaped into the cavity of the abdomen. The surrounding parietes were also much thickened, but presented no well-marked appearance of recent inflammation or ulceration. The other organs, with the exception of the liver, which presented the pathological condition of incipient cirrhosis, appeared healthy.

*Remarks.* This case derives especial interest from the obscurity of the diagnosis; and though I had inferred that the *liver* was probably the seat of the disease, the absence of tenderness and continued pain in the right hypochondrium and at the scrobiculus cordis, the want of an icteric tinge in the conjunctiva, and the regular bilious evacuations, seemed to preclude the possibility of an obstruction of the gall ducts, nor in any way determined the precise nature of the disease.—*Boston Med. & Surg. Journal.*

